# T cell specific and non specific tolerance mechanisms in peanut oil immunotherapy in peanut allergic subjects

**DOI:** 10.1186/2045-7022-1-S1-O34

**Published:** 2011-08-12

**Authors:** Amit Singh, Brittany Weldon, Grace Yu, Sue Neale-May, Tessa Hunter, Kari Nadeau

**Affiliations:** 1 Stanford University, Pediatrics Department, Stanford Medical School, Stanford, USA

## Rationale

Our main focus in the laboratory is to understanding the mechanisms of how oral immunotherapy (OIT) improves outcomes in patients. In this study, we aimed to determine if OIT is specific only to the food allergens administered in OIT or also to other offending allergens (i.e. "bystander effect"). To accomplish our aim, we studied T cell reactivity (i.e. proliferation assays) and T cell specificity (i.e. tetramer assays with peanut peptides) in both effector T cell (CD4+CD25neg) or Teff and regulatory T cell (CD4+CD25hiFoxp3+) or Treg subsets.

## Methods

A phase I trial of oral immunotherapy for peanut allergy was approved by the Stanford IRB and conducted according to ICH guidelines. All subjects (n=24) had a moderate to severe clinical reactions to peanut ingestion, peanut-specific IgE ≥ 15 kU/L, and a positive skin-prick test. Subjects underwent an initial dose escalation to a maximum dose prior to initiating daily home dosing with q2wk dose. Blood samples were collected at baseline and throughout the course of the treatment. T cells were magnetically purified and tested for specificity to peanut epitopes, other offending food allergens for the patient, and a control antigen. Basophil reactivity was also assessed using a whole blood assay. Clinical assessments were performed using Bock's criteria.

## Results

Twenty-four subjects, ages 5 to 45, have received 3777 doses. Teff cell proliferation (using 3H thymidine assays) was optimally suppressed by autologous Treg when activation occurred using antigen presenting cells (APCs) loaded with peanut, rather than loaded with other offending food allergens (i.e. milk). Basophil reactivity (as determined by CD203c) was downregulated during the course of OIT to both peanut and other offending allergens.

## Conclusions

We report on our immune monitoring results to date at Stanford Hospital and Clinics with a phase I peanut oral immunotherapy study using published methods. With T cell monitoring, we were able to show downregulation of specific peanut-induced proliferation and that this downregulation was mediated by Treg specific to peanut epitopes.

